# Nucleotide Excision Repair in Cellular Chromatin: Studies with Yeast from Nucleotide to Gene to Genome

**DOI:** 10.3390/ijms130911141

**Published:** 2012-09-07

**Authors:** Raymond Waters, Katie Evans, Mark Bennett, Shirong Yu, Simon Reed

**Affiliations:** Institute of Cancer and Genetics, School of Medicine, Cardiff University, Heath Park, Cardiff CF14 4XN, UK; E-Mails: evansKE3@cardiff.ac.uk (K.E.); bennettMR1@cardiff.ac.uk (M.B.); yuS@cardiff.ac.uk (S.Y.); reedSH1@cardiff.ac.uk (S.R.)

**Keywords:** global genome nucleotide excision repair, chromatin, *Saccharomyces cerevisiae*, UV-induced cyclobutane pyrimidine dimers, histone acetylation, gene studies, genome-wide studies

## Abstract

Here we review our development of, and results with, high resolution studies on global genome nucleotide excision repair (GGNER) in *Saccharomyces cerevisiae*. We have focused on how GGNER relates to histone acetylation for its functioning and we have identified the histone acetyl tranferase Gcn5 and acetylation at lysines 9/14 of histone H3 as a major factor in enabling efficient repair. We consider results employing primarily *MFA2* as a model gene, but also those with *URA3* located at subtelomeric sequences. In the latter case we also see a role for acetylation at histone H4. We then go on to outline the development of a high resolution genome-wide approach that enables one to examine correlations between histone modifications and the nucleotide excision repair (NER) of UV-induced cyclobutane pyrimidine dimers throughout entire genomes. This is an approach that will enable rapid advances in understanding the complexities of how compacted chromatin in chromosomes is processed to access DNA damage and then returned to its pre-damaged status to maintain epigenetic codes.

## 1. Introduction

The seminal research of Smerdon [1–3] and Thoma [3,4], and the fact that *in vitro* core nucleotide excision repair (NER) factors could not repair DNA damage in chromatin [5], showed that chromatin influenced how NER operated in eukaryotic cells. Results from Smerdon and colleagues showed that in mammalian cells there was a role for histone acetylation in NER [1,2] and those from the Thoma group, primarily working with yeast, indicated that linker DNA between nucleosomes was repaired more rapidly than the core DNA residing within nucleosomes [3,4]. These experiments showed that chromatin structure impinged on the efficiency of NER.

In light of these data we focussed our research so as we could build on these observations. We began by developing technologies to address specific questions related to how NER operates on chromatin in *Saccharomyces cerevisiae* exposed to DNA damaging agents. *S. cerevisiae* was selected because it is a genetically tractable organism, it has NER genes which bear considerable homology to those in humans, and there are cells available that harbour a broad range of mutations in genes with roles in NER and chromatin modifications [5]. We used UV irradiation at 260 nm to damage DNA and we concentrated on the repair of cyclobutane pyrimidine dimers (CPDs). These are the most frequent lesions induced by this treatment, their change in frequency has been used regularly to examine how NER operates, and they can be detected by enzymes that cut DNA at the CPDs or via antibodies to precipitate DNA that contains them [5].

Initially we developed an approach to quantify the frequency of individual CPDs at any location in any selected sequence. This was first developed with *Escherichia coli* [6] and subsequently modified for use with yeast [7]. The approach employed probes to separate from the rest of the yeast genome specific yeast sequences isolated as individual single strands. CPDs within these sequences were detected by virtue of cutting with a CPD-specific DNA glycosylase so reducing the migration of the labelled strands in polyacrylamide DNA sequencing gels. We could not only measure the frequency of the induction of individual CPDs in the sequence of choice but also the changes in their frequency during DNA repair [6–14].

We next adapted the method for the high resolution footprinting of yeast nucleosomes [15]. Here the accessibility of the DNA in chromatin to cutting by Mnase was used and sequences isolated and analysed as for CPD detection. We were able to map nucleosome positions to within a few base pairs in the selected sequences (see later). This development enabled us to use IP and RT-PCR to examine covalent modification of histones in specific placed nucleosomes in order to see if they are modified after DNA damage and to identify enzymes with roles in this [16–20].

It also meant that we could identify restriction sites within nucleosome cores to analyze their accessibility in chromatin before and after DNA damage [16,19]. This accessibility can depend on Swi/Snf factors which use ATP to remodel chromatin and which can move nucleosomes in cis or trans [21,22].

These approaches enabled us to discover that UV induced histone H3 acetylation at certain repressed yeast genes occurs via the Gcn5 Histone acetyltranferase (HAT) [11,16–20]. *Gcn5* is essential for efficient NER at some yeast genes and this role is independent of Gcn5s’ role in transcription [16]. Details of these experiments are described and discussed in the following section.

NER has two sub-pathways, transcription coupled NER (TC-NER) and global genome NER (GG-NER) [5]. TC-NER uniquely operates on the transcribed strand of transcriptionally active genes and GG-NER operates on the transcribed strand as well as on the non transcribed, plus on all transcriptionally silent regions of the genome. These sub-pathways differ only in the means of detecting DNA damage; TC-NER relies on a RNA polymerase stalled at a CPD signalling that the CPD requires repair [23,24], whereas GG-NER in *S. cerevisiae* relies on a GG-NER-specific complex that is composed of Rad16/Rad7 and the autonomously replicating sequence binding factor I (Abf1) [25–27]. Following damage recognition the subsequent steps in NER appear to be the same. In this review we will focus on GG-NER.

Rad16 is a member of the SWI/SNF super-family of chromatin remodelling factors [28]. This superfamily of proteins exhibits ATPase activity that is stimulated by DNA or chromatin [29,30], and all SWI/SNF-like proteins generate superhelical tension in linear DNA fragments via a DNA translocase activity associated with their ATPase function [31,32]. The generation of superhelicity in DNA is a common mechanism of SWI/SNF-like chromatin remodelling complexes for altering chromatin structure [31]. We recently reported that a Rad7 and Rad16 containing protein complex also has DNA translocase activity. However, it is unable to slide nucleosomes unlike some SWI/SNF superfamily complexes [33]. Rad7 and Rad16 form a stoichiometric complex [26,34] that binds damaged DNA in an ATP-dependent manner [35]. Rad7 is part of an E3 ligase complex that ubiquitinates Rad4 a core yeast NER protein that binds to damaged DNA [36]. This ubiquitination of Rad4 in response to UV specifically regulates NER via a pathway that requires *de novo* protein synthesis and it directly influences NER and UV survival [36]. Abf1 was originally identified for its ability to bind DNA at a variety of origins of DNA replication, as well as the silencing loci *HML* and *HMR* [37]. A plethora of literature subsequently identified Abf1 to bind within the upstream activating sequences (UASs) of a large array of gene promoters. It is now well established that Abf1 is an abundant, essential, global site-specific DNA binding protein [38]. A role for Abf1 in GGR came from the observation that the protein co-purifies with Rad7. In the absence of UV damage, Abf1 forms a stable heterotrimeric complex with Rad7 and Rad16, termed the GGR complex and approximately one third of cellular Abf1 is predicted to be in a complex with Rad7 and Rad16 [26].

Here we will describe our studies on the GG-NER of CPDs from specific cellular genes and subsequently throughout the entire yeast genome. We have uncovered some covalent histone modifications that are linked to efficient GG-NER, determined how these relate to Swi/Snf activity and determined that they are facilitated by the GG-NER specific complex of Rad16/Rad7 and Abf1. From these observations we will propose a model to suggest how the GG-NER complex operates to govern the removal of UV-induced DNA damage from yeast chromosomes.

## 2. Results and Discussion

**Figure 1:**
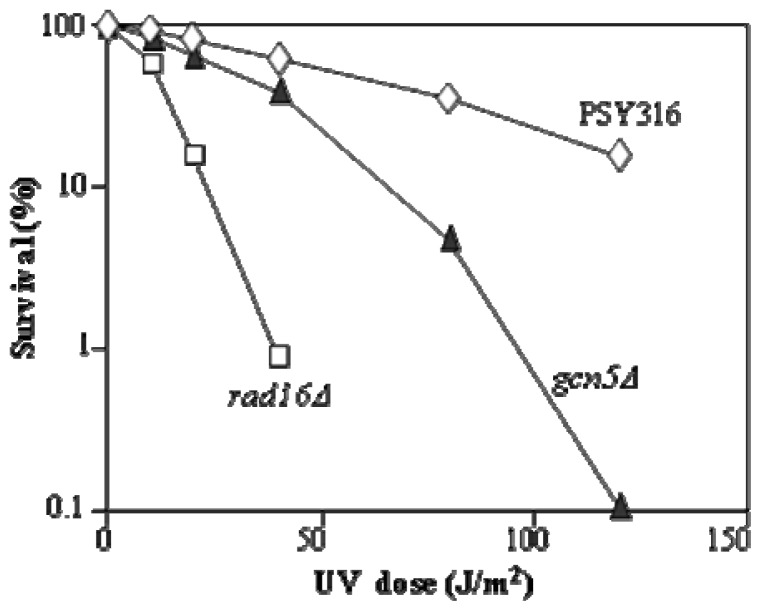
Exponential phase cells with either no deletions or deletions of *RAD16* or *GCN5* were treated without UV or with a range of UV 260 nm doses. Cells were then plated out on Yeast Extract Peptone Dextrose (YPD) plates. Colonies were counted after three days growth at 28 °C and the percent survival calculated compared to the un-irradiated sample [11].

The first observation we made with respect to unravelling how NER requires chromatin change to operate effectively was that the absence of the yeast HAT Gcn5 conferred some UV sensitivity [11]. As seen from Figure 1 the UV sensitivity is slight and less than that conferred by the absence of the GG-NER specific protein Rad16. This observation with the *GCN5* deletion mutant could have been due to a number of events, including changes in the transcription of NER genes, or cell cycle modifications, so we went on to determine why these cells were UV sensitive.

We showed that this UV sensitivity was not due to an overall defect in NER.

**Figure 2:**
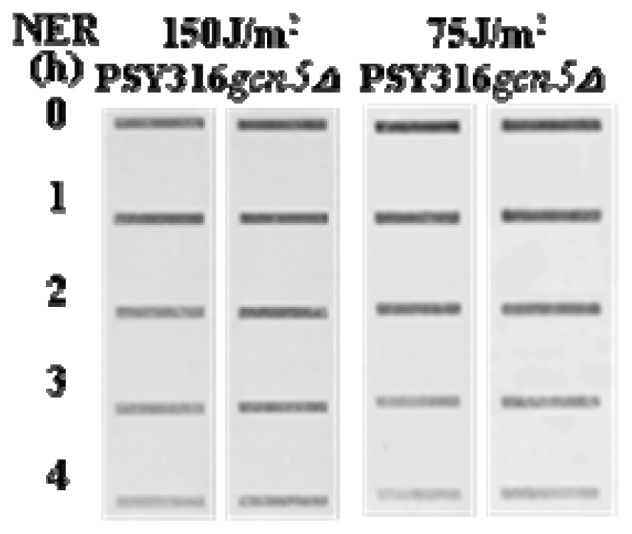
Cells with or without Gcn5 were irradiated with 75 or 150 J/m^2^ of UV at 260 nm. DNA was sampled either immediately or after the cells had been incubated in YC medium for various repair times. DNA was purified and aliquots containing equal amounts of DNA were subjected to slot blotting with an antibody specific to cyclobutane pyrimidine dimers (CPDs) [11].

**Figure 3:**
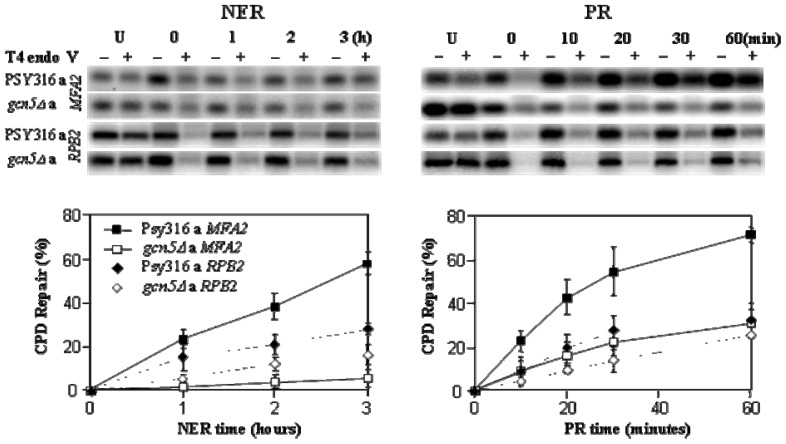
Exponential phase *a* mating type cells either with or without Gcn5 were UV irradiated with 70 J/m^2^ of UV at 260 nm and either sampled immediately or after 1, 2 or 3 h post UV in YC medium for nucleotide excision repair (NER) to occur. DNA was purified and incubated with (+) or without (−) a CPD specific DNA glyscosylase. The DNA was run on alkaline agarose gels before Southern blotting to analyse the non-transcribed strand and hence the frequency of GG-NER. Loss of signal is indicative of CPDs and the return of signal indicative of their repair. The return of signals is plotted graphically as % repair [11].

Figure 2 shows slot blots of CPD incidence at various times after UV exposure in cells with or without Gcn5. There is no discernable differences in NER from the genome overall. However, when we examined the changes in CPD frequency in *MFA2 versus* the *RBP2* genes there was much less efficient NER at the *MFA2* gene in the absence of Gcn5 but no significant change in NER at *RPB2*. Figure 3 shows Southern blots to detect CPDs in the *MFA2* and *RPB2* genes. There is a clear and statistically significant defect in NER at *MFA2* but not at *RPB2.* These data show that NER is indeed influenced, but only at some locations within the genome.

Our early studies with *MFA2* had been developed with a view to examining DNA repair at nucleotide resolution using a method that we had developed [6,7]. *MFA2* is a mating type specific gene that is transcriptionally active in *a* cells but which is repressed in α cells. The HAT Gcn5 plays a role in governing the transcription of *MFA2* in *a* cells and in the absence of Gcn5 the transcription of *MFA2* is reduced to about 30% of normal [11]. By studying *MFA2* we could study TC-NER *versus* GG-NER in separate cellular environments where transcription of this gene was present or absent.

**Figure 4:**
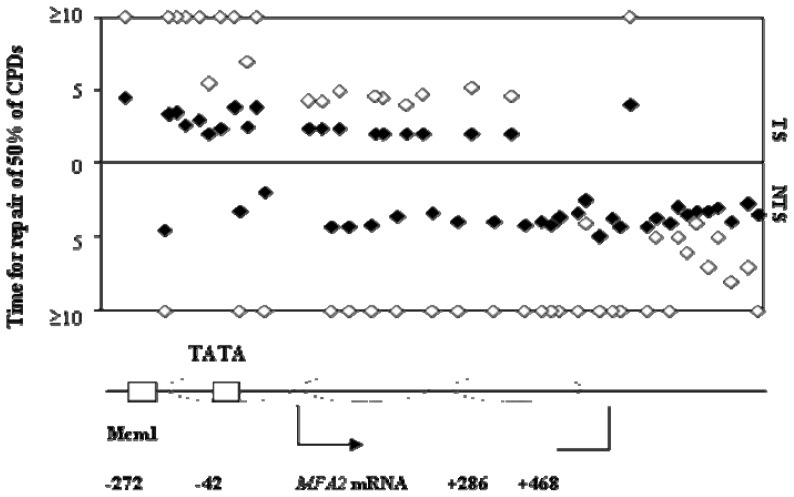
Exponential phase *a* cells with (closed symbols) or without Gcn5 (open symbols) were treated with 150 J/m^2^ of UV at 260 nm and the DNA sampled either immediately or after various repair times. The DNA was analyses for the frequency of CPDs as [7]. The data are expressed as the time (h) taken to remove half of the CPDs induced at given dipyrimidine sites throughout the *MFA2* regulatory region and coding sequence. The top half of the Figure shows the data for the transcribed strand and the bottom half the data for the non-transcribed strand. Nucleosome positions when the gene is repressed are denoted by ovals and the Mcm1 binding site, the TATA box plus the start and stop sites for transcription are given.

We investigated the NER of UV induced CPDs from the transcriptionally active *MFA2* in a cells [11]. Figure 4 shows the half life of CPDs at nucleotide resolution after *a* mating type yeast cells were UV irradiated with 150 J/m^2^; this dose induced on average one CPD at *MFA2* in about 30% of the cell population. We observed enhanced repair for the transcribed strand (TC-NER plus GG-NER) as opposed to non-transcribed regions where only GG-NER would operate. There is a clear reduction in the half life of CPDs in the *GCN5* mutant in regions that are subjected to TC-NER and GG-NER and in those that are uniquely subjected to GG-NER.

We next decided to examine the repair of CPDs in α cells where we knew that *MFA2* was transcriptionally repressed. Just as in *a* cells without Gcn5, these α cells had a defect in GG-NER at *MFA2*; it was reduced to about 30% of the efficiency seen in cells possessing this HAT [16].

**Figure 5:**
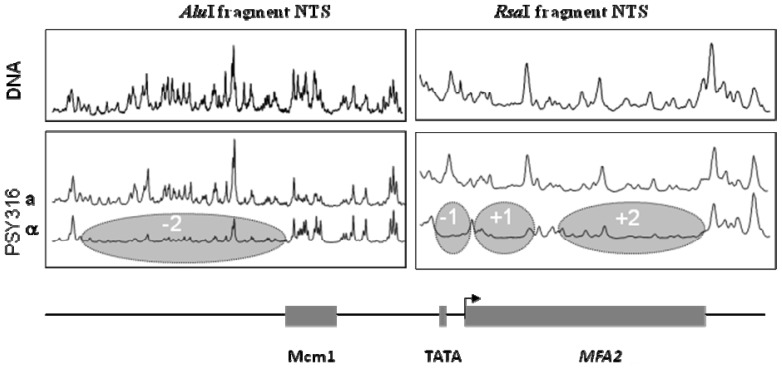
Chromatin from a or *a* mating type cells was incubated with Mnase and processed as in [15]. Scans are shown of band intensities on agarose gels; the higher the peaks then the more efficient the cutting by Mnase. The absence or reduction in peaks in α cells as opposed to in *a* cells relates to the positioning of 4 nucleosomes as indicated; each occupies about 147 bp of the *MFA2* sequence shown underneath.

Hence we were in a position whereby we could examine the nuances of GG-NER at nucleotide resolution in genes of choice. However, we wished to relate these events to changes in chromatin that were linked to enabling efficient GG-NER. Therefore we needed to examine the nature of any nucleosomes at *MFA2* in both *a* and α cells so as we could relate our repair data to the presence of nucleosomes, their positions, or their absence and their modifications. We reasoned that we could adapt our method to detect CPDs at nucleotide resolution in order to determine nucleosome positions at *MFA2.* This was undertaken not by examining the results of cutting with a CPD specific enzyme as for DNA damage analyses, but by examining the results of cutting DNA in chromatin with Mnase; a standard way of determining nucleosome positions at low resolution [38]. To afford a high resolution analysis we then purified DNA fragments of choice exactly as we did for the same fragments when analysing DNA damage. We then radiolabelled and ran those fragments on alkaline agarose gels to identify any MNase protected regions at *MFA2*. If protected regions equivalent to lengths of about 147 base pairs are identified then this is an indication of the presence of a nucleosome in that region [15]. As shown in Figure 5 there were no fixed nucleosomes in chromatin from *a* mating type cells where *MFA2* is transcriptionally active but in α mating type cells where the gene is repressed there are four positioned nucleosomes in the chromatin. Two of these nucleosomes reside in the *MFA2* regulatory region, either side of the Mcm1 binding site with that nucleosome labelled as −1 being over the TATA box. Then there are 2 positioned nucleosomes within the *MFA2* coding region of this relatively small gene.

This nucleosome mapping enabled us to employ immunoprecipitation and RT-PCR to examine histone acetylation at these nucleosomes pre- and post-UV. It also meant that we could identify restriction sites at the cores of the fixed nucleosomes in α mating type cells and examine their accessibility pre- and post-UV in these cells.

**Figure 6:**
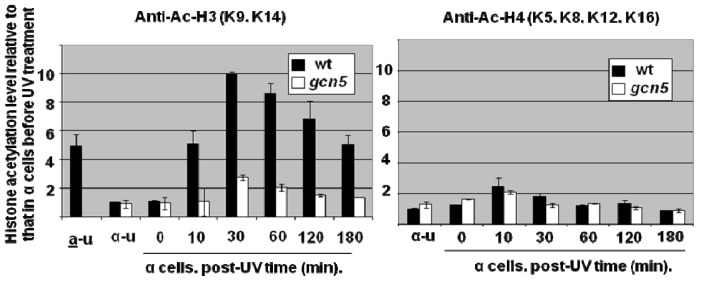
Relative plots pre- and post- UV of histone H3 acetylation at lysines 9/14 (**left** hand graph) and histone H4 (**right** hand graph) in the 2 nucleosomes of the *MFA2* regulatory region [16]. Data are in unirradiated a-cells where *MFA2* is transcriptionally active, and in α cells where it is transcriptionally repressed and where cells were either unirradiated or given 150 J/m^2^ and then incubated for various times after UV.

In α mating type cells possessing the HAT Gcn5 there was a UV-induced increase in histone H3 acetylation at lysines 9/14 for the two nucleosomes in the *MFA2* regulatory region of the transcriptionally repressed gene; most of this was mediated by Gcn5 (Figure 6). We have not examined whether this acetylation extends to the nucleosomes in the coding sequence of the gene. At the nucleosomes within the *MFA2* regulatory region there is little increase in acetylation at histone H4 [16].

**Figure 7:**
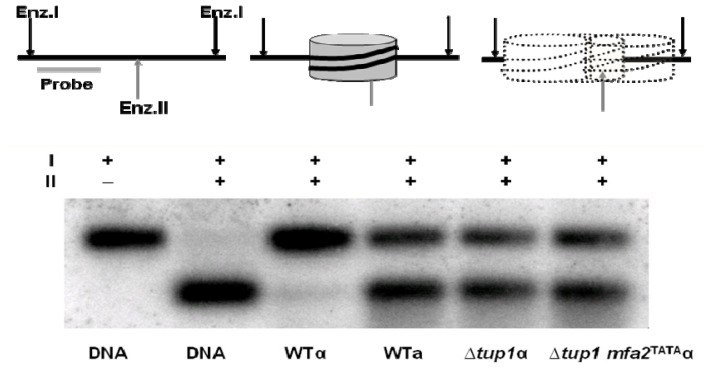
A restriction enzyme accessibility assay to examine the relationship between nucleosomes and core DNA. Chromatin was digested with either Restriction Enzyme I (*Hae*III) alone or with HaeIII plus Enzyme II (RsaI). HaeIII sites occur either side of the −2 positioned nucleosome of the repressed *MFA2* gene, whereas the RsaI site resides within the core DNA of this nucleosome. DNA from these digests was run on agarose gels and probed with a radiolabelled sequence as indicated and described in [16].

Hence Gcn5 had a role in facilitating efficient GG-NER at *MFA2*. This result is not restricted to *MFA2* and we also observed the same effects at *MET16* when it was transcriptionally repressed or transcriptionally active [18]. Importantly, this role of Gcn5 in GG-NER at *MFA2* was totally outside of its role in transcription in *a* mating type cells because we showed that this H3 acetylation after UV in these cells was not related to any aberrant transcriptional activation of this repressed locus [16]. Unlike in un-irradiated *a* mating type cells, there was no detectable recruitment of TATA box binding protein to the *MFA2* TATA box in UV treated α cells, nor was there any detectable *MFA2* transcript in these cells. At the same two fixed nucleosomes of the *MFA2* regulatory region in α mating type cells we examined in chromatin the accessibility of restriction sites within the nucleosomal core DNA [16]. Figure 7 describes the logic behind this assay and provides images of gels showing the changes in fragment size in DNA from the chromatin for nucleosome −2. The assay was performed on cells of both mating types and in cells where the Tup1 repressor which represses *MFA2* was deleted [38,39]. This deletion de-represses the gene and creates a hyperacetylated histone H3 state at lysines 9/14 in the nucleosomes of the regulatory region. We additionally examined events in *TUP1* deleted cells where there was also a mutated TATA box to prevent transcription; so here we had created chromatin that was more accessible and hyperacetylated, but in the absence of *MFA2*’s transcription and any potential TC-NER.

This assay reveals that the *Rsa*I site is poorly accessible in the chromatin from α mating type cells where *MFA2* is repressed but that this accessibility is dramatically increased when the gene is transcriptionally active in *a* mating type cells or when the Tup1 repressor is absent in α cells, irrespective of the ability to transcribe the gene.

**Figure 8:**
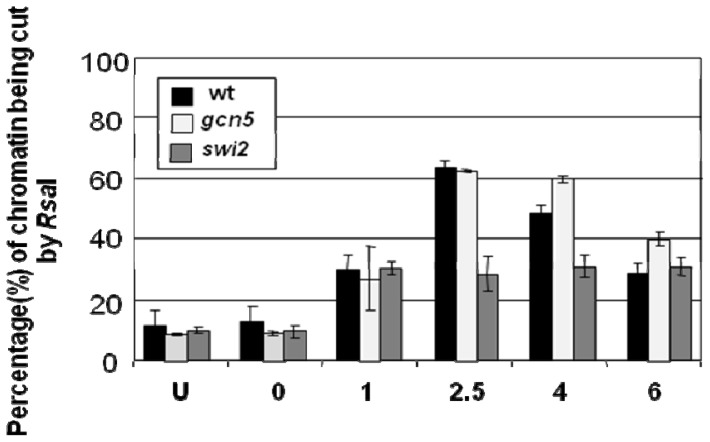
The accessibility in chromatin of the *Rsa*I restriction site within the core of the −2 nucleosome in the *MFA2* regulatory region.

Figure 7 showed that without UV the *Rsa*I site is accessible in *a* mating type cells but not in α cells. However, as seen in Figure 8, after UV the site in α mating type cells becomes accessible and this is partly dependant on Swi2 [16].

**Figure 9:**
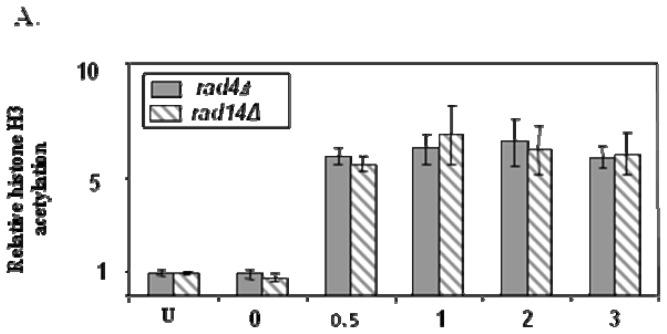

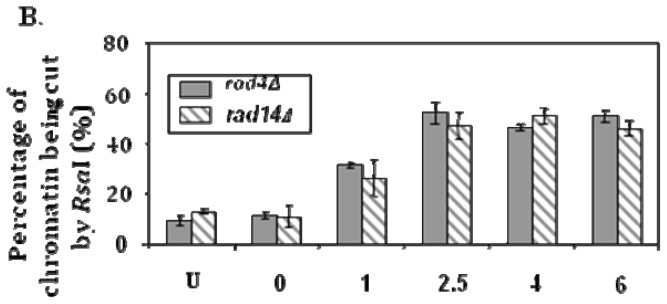
UV induced histone H3 acetylation (**A**) and the accessibility of the *Rsa*I restriction site (**B**) within the *MFA2* regulatory region post UV for NER defective *rad4* and *rad 14* mutants [16].

Surprisingly both this event and the UV induced histone acetylation occurred in *rad4* and *rad14* NER defective cells. However, in these instances neither event decreased as in NER-competent cells and where repair occurred (Figure 9) [16]. This indicates that functional NER is not required to trigger these events but that it is required to restore these events to their pre-DNA damage levels. The roles of SWI/SNF and INO80 in NER have been researched in greater depth by the research groups of Smerdon and McHugh, so further details can be obtained from their reviews in this issue.

**Figure 10:**
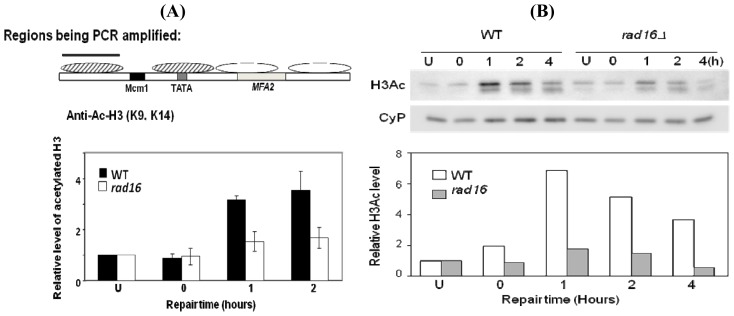
Exponential phase repair competent cells or those with *rad16* deleted were un-irradiated (U) subjected to 70 J/m^2^ of UV and either sampled immediately (0) or at various repair times after UV (1, 2, 4 h). The Relative levels of histone H3 acetylation at lysine 9/14 was measured at the −2 nucleosome of the *MFA2* gene (**A**) or in total chromatin via the Western blots shown and quantified in (**B)** [19].

The result above poses the question as to what factors govern this UV-induced change in chromatin that facilitates efficient GG-NER? Thus we went on to investigate if any proteins with roles in GG-NER could govern these UV-induced parameters. We were intrigued to discover that the UV-induced histone H3 acetylation required the GG-NER specific protein Rad16 (Figure 10) [19].

This requisite existed for events at *MFA2* and for those throughout the genome. Hence Rad16 had a genome-wide role in governing UV-induce histone H3 acetylation [19]. At this stage it was unclear as to how Rad16 mediated this acetylation. Interestingly, in cells where the repressor Tup1 [39,40] was absent there was pre-UV H3 hyperactylation at the *MFA2* regulatory region and at other loci where Tup1 governed H3 acetylation. Rad16 was not required for GG-NER at these sequences in this *Tup1* mutant [20].

This hyperacetylation was much reduced both without UV, and after UV by the combined absence of Rad16 and Gcn5 [20]. This suggested that there were intimate links between Rad16 activity and that of Gcn5 during GG-NER and that the activity of these proteins jointly governed the extents of GG-NER.

**Figure 11:**
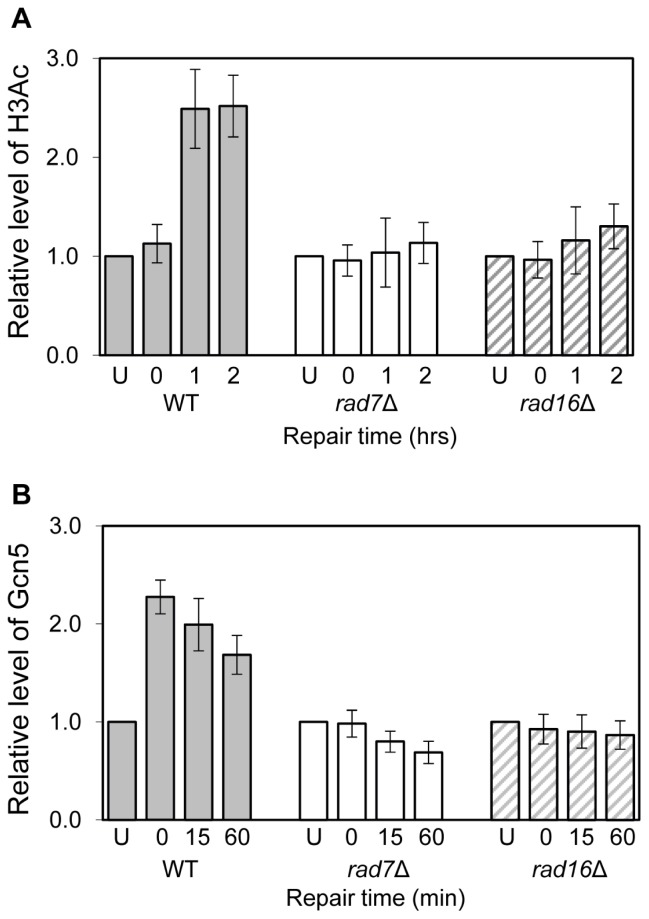
Relative levels pre- and post- UV (70 J/m^2^) of Histone H3 acetylation and the HAT Gcn5 within the promoter region of *MFA2* of GG-NER competent cells, and the *rad7* or *rad16* GG-NER defective mutants [20].

We undertook further experiments designed to unravel the role of Rad16. These revealed that post UV both Rad16 and Rad7 governed the occupancy at *MFA2* of the HAT Gcn5 [20] (Figure 11).

**Figure 12:**
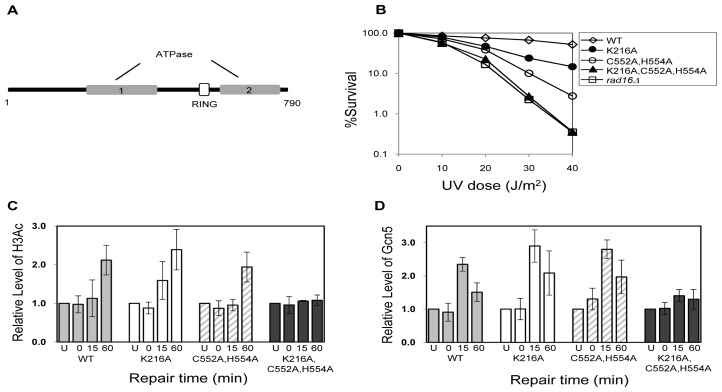
(**A**) The domain structure of Rad16; (**B**) UV survival curves of the strains indicated; (**C**) Histone H3 acetylation at the MFA2 promoter. ChIP analysis of Histone H3 acetylation (H3Ac) was performed using H3Ac (Lys 9 and Lys 14) antibody. U: untreated samples; 0: cells received 100 J/m^2^ of ultraviolet without repair; 15 and 60: cells were irradiated with UV and then were allowed to repair in medium for the times indicated. Acetylation level shown as the fold change relative to unirradiated cells; (**D**) The occupancy of Gcn5 at the MFA2 promoter. ChIP was performed with anti-myc antibody. Gcn5 binding is presented as the fold change relative to untreated cells. [20].

**Figure 13:**
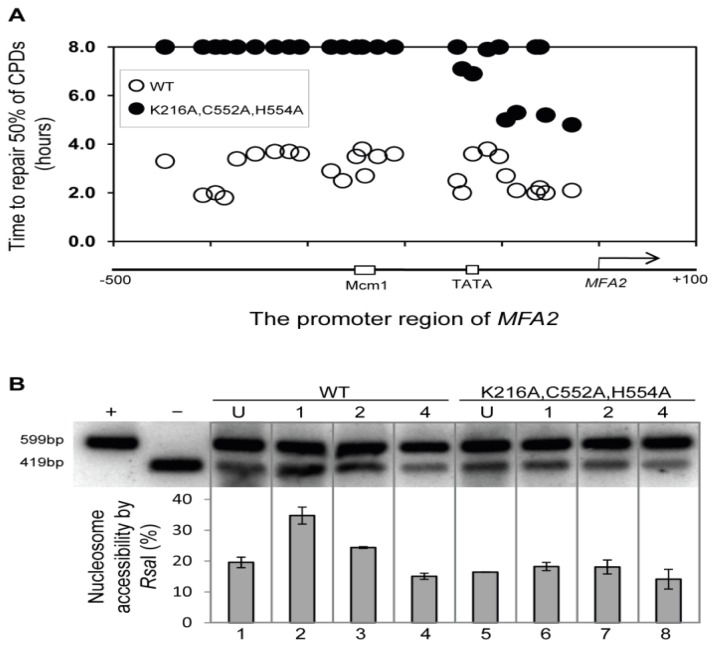
Repair of CPDs at the MFA2 promoter. Time to remove 50% of the initial CPDs (T_50%_) at given sites. T_50%_ of a single CPD or a clustered group of CPDs with a similar repair rate was calculated as described previously [11]. (**A**) The T_50%_ of unrepaired CPDs (T_50%_ ≥ 8 h) were represented at the 8 h level on the graph; (**B**) Southern blot analysis of *Rsa*I accessibility to the *MFA2* promoter N-2 nucleosomal DNA [20].

To examine the role of Rad16 further we decided to create mutants at each of its two active sites, namely the ATPase domain and the site for E3 ubiquitin ligase activity associated with the C3HC4 RING domain that is embedded within the ATPase domain. We mutated either each one alone or both of these in combination [20]. Creation of the point mutations within the Rad16 ATPase domain (K216A), RING-finger domain (C552A,H554A) and double mutation of ATPase and RING-finger domains (K216A,C552A,H554A), resulting in the amino-acid substitutions, lysine 216 to alanine, cysteine 552 to alanine and histidine 554 to alanine. This approach revealed that mutations in both its ATPase site and its ring domain were required to totally inactivate the recruitment of Gcn5 and the subsequent Histone H3 acetylation at lysines 9/14 (Figure 12). Figure 13 shows how the repair of CPDs within the *MFA2* regulatory region is reduced in the ATPase and ring domain double mutant (A) and how this impinges on the accessibility of the RsaI site residing within the core of nucleosome −2 (B).

**Figure 14:**
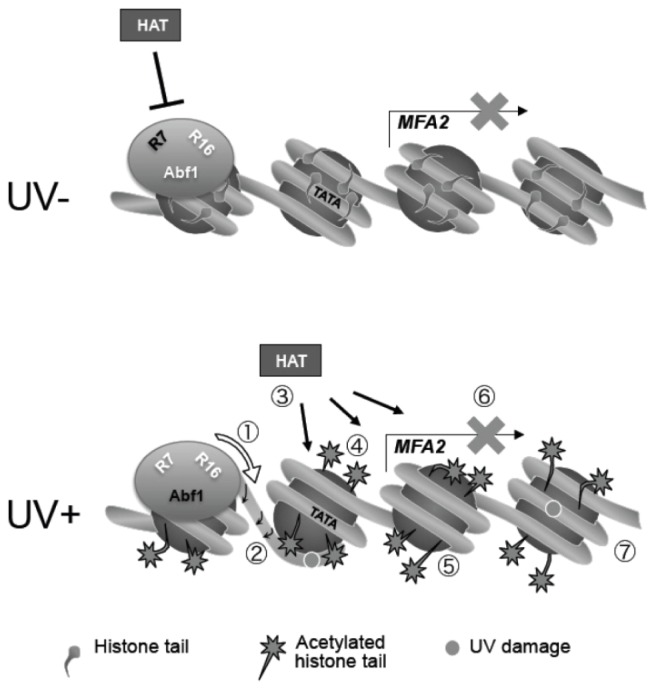
Model for UV induced chromatin remodeling during GG-NER. **Top** panel: In the absence of UV, basal levels of histone acetyl transferase occupancy is observed, histone H3 tails remain unacetylated and chromatin is repressed. **Lower** Panel: Following UV the DNA translocase (**1**) and E3 ligase (**2**) activities of Rad16 in the GG-NER complex promote increased histone acetyl transferase occupancy (**3**) and histone H3 acetylation (**4**) that drives chromatin remodeling (**5**). Failure of the GG-NER complex to slide nucleosomes may prevent transcription factor binding explaining the continued repression of MFA2 transcription (**6**) despite chromatin remodeling. GG-NER dependent chromatin remodeling promotes efficient lesion removal [7].

These data have shed some considerable insight in how GG-NER operates on repressed chromatin and they have led us to propose the model shown in Figure 14. In wild type cells in the absence of UV damage (Figure 14 upper panel), only basal levels of Gcn5 occupancy at the promoter region of *MFA2* are observed, consequently the histone H3 acetylation status is maintained at a low level, and chromatin remains in a closed configuration preventing gene transcription from *MFA2*. Following UV irradiation (Figure 14 lower panel), Rad7 and Rad16 dependent increased occupancy of the histone acetyl transferase Gcn5 (Figure 14(3)) results in elevated levels of histone H3 acetylation (Figure 14(4)). This promotes an open chromatin structure at *MFA2* (Figure 14(5)). Our results show that this is achieved via the dual action of the DNA translocase (Figure 14(1)) and E3 ligase (Figure 14(2)) activities associated with the ATPase and RING domains of the Rad16 component of the GG-NER complex. We recently showed that the GG-NER complex exhibits DNA translocase activity associated with the ATPase domain of Rad16 [33]. This activity generates superhelical torsion in DNA necessary for excision of DNA damage during GG-NER *in vitro*. In the same study we also reported that the GG-NER complex is not able to slide nucleosomes in an *in vitro* assay, unlike many SWI/SNF chromatin remodelling complexes. Given these results, we hypothesise that the DNA translocase activity of the GG-NER complex specifically modifies chromatin structure to facilitate access of the Gcn5 histone acetyl transferase, which subsequently promotes an open chromatin structure necessary for efficient GG-NER via increased histone H3 acetylation in the region. We reported previously that during GG-NER at *MFA2*, the gene remains repressed. We suggest that failure of the GG-NER complex to slide nucleosomes at the promoter of *MFA2* in response to UV prevents access to critical transcription initiation sites such as the TATA box, which precludes the sequence specific binding of key transcription initiation factors. In this way, the GG-NER complex can promote specific changes in chromatin structure required for efficient GG-NER (Figure 14(7)), while at the same time preventing unregulated gene transcription (Figure 14(6)). Our results also reveal the importance of the RING domain of Rad16 in promoting chromatin remodelling necessary for efficient GG-NER. This suggests that the E3 ubiquitin ligase activity of the GG-NER complex also plays a role in chromatin remodelling necessary for repair.

Much of our focus has been on the role of histone H3 acetylation in GG-NER, but acetylation at histone H4 also goes up after UV. As we saw in Figure 6, there was little increase in histone H4 acetylation post UV within the *MFA2* gene, so where does histone H4 acetylation play a role? Our research on how NER operates in repressed and non-repressed sub-telomeric sequences of *S. cerevisiae* has thrown some light on this issue [41].

To examine GG-NER at subtelomeric regions we employed and modified constructs from the Louise laboratory where the *URA3* gene had been inserted into either transcriptionally active (NRE) or repressed (RE) subtelomeric regions of the yeast genome [42]. We needed to create strains with a single URA3 sequence to employ our high resolution DNA damage detection technology so we removed the mutated endogenous URA sequence from its natural location in the original strains [41].

**Figure 15:**
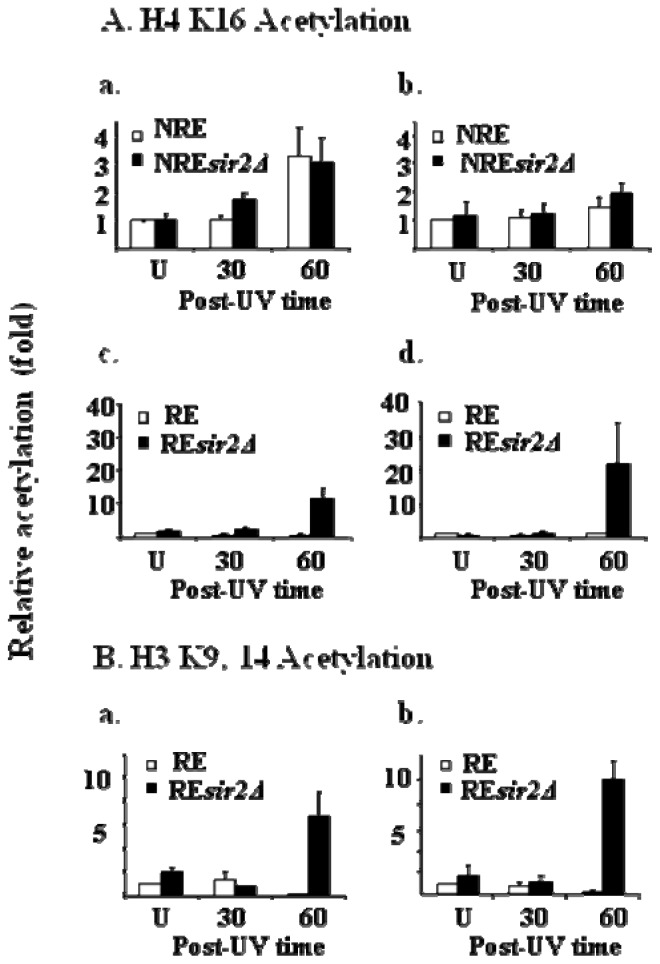
Histone acetylation at the coding region of the subtelomeric URA3 in response to UV irradiation. The histone H4 K16 (**A**), H3 K9, K14 (**B**) are represented as a relative level to that in the RE before UV treatment. The acetylation level of H3 K9, K14 and H4 K16 was normalized against histone H3 loading data. **a.** in the region from +222 to +369; **b.** in the region from +540 to +669 (41). Post UV time is in minutes.

These reconfigured *URA3* strains enabled us to examine the repair of CPDs in unique identical subtelomeric sequences under either transcriptionally active or repressed circumstances. We found that NER is significantly more efficient in the NRE than the RE. At the NRE UV radiation stimulates both histones H3 and H4 acetylation (Figure 15). These modifications occur regardless of the presence of the Sir2 Histone deacetylase. On the other hand, at the repressed subtelomere, where GG-NER is much less efficient, UV radiation is unable to stimulate histone H4 or H3 acetylation in the presence of Sir2. In the absence of Sir2 these UV-induced modifications are detected [42] and Figure 15B shows the histone H3 data; these result in a significant increase in NER efficiency in the region. Interestingly, these experiments reveal that not only as there different spectra for histone acetylations associated with DNA damage, but that there are instances in the yeast genome where the maintenance of the existing chromatin structures dominates over the action of chromatin modifications associated with efficient NER.

Thus our data indicate that the types of histone acetylation induced by UV can vary depending on the genomic location of the DNA damage. We also know that chromatin structure varies through genomes and that the repair of CPDs is also variable. Thus studies with either a single model gene or a few model genes have a limit to the information that they divulge. None of the approaches employed in the studies that enabled us to unravel the facts described above could enable us to examine repair events throughout genomes at a high resolution; they are simple far too laborious to undertake this exercise. As a result, and in order to identify variations in repair rates and to reveal any correlation of repair with changes in chromatin structure genome-wide we developed further technology. We reasoned that an approach for analysing entire genomes at high resolution for DNA repair would enable one to examine the global influence of factors on repair. For example, the recruitment of repair proteins to DNA damage in chromatin and how this relates to the chromatin modification factors that facilitate this for DNA repair.

**Figure 16:**
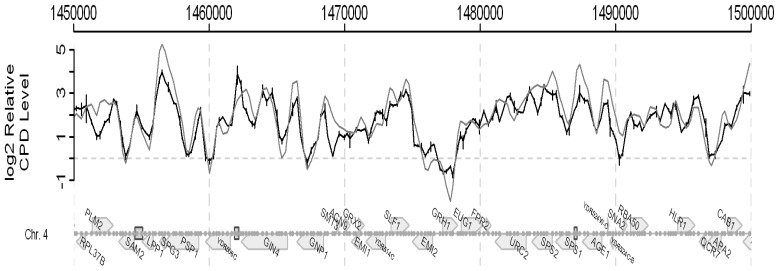
The CPD distribution in a part of Chromosome 4. Black: CPD levels detected by the CPD antibody; Grey: theoretical CPD distribution. The bars represent standard deviations. For more detail see [45].

DNA microarrays were developed decades ago for whole genome transcription profiling. The combination of these and chromatin immunoprecipitation, namely ChIP on chip, was an extension that enabled the identification of the binding sites of DNA-binding proteins and the covalent modifications to nucleosomes on a genome-wide basis [43,44]. We built on this technology by developing a genome wide ChIP on Chip approach that employs microarrays to monitor UV-induced DNA damage (CPDs) and its repair [45]. Consequently, this method means that we can identify the UV-induced changes in chromatin and the chromatin modifications that facilitate repair throughout an entire genome. The method is described in detail in our paper by Teng *et al.* [45]. A snapshot of the frequency of CPD induction in part of the yeast genome is shown in Figure 16 and the data for the entire yeast genome can be seen in the above paper [45].

**Figure 17:**
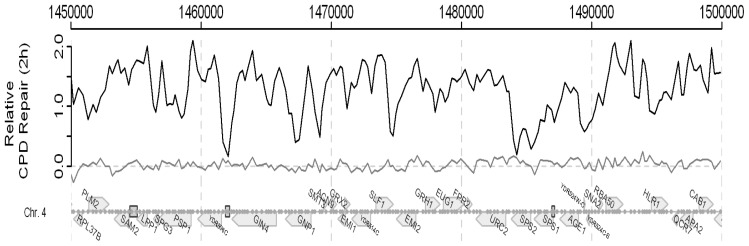
Relative CPD repair rate in a part of chromosome 4. Black: NER proficient cells; Grey: NER deficient *rad4* cells. For more details see [45].

In this Figure we plot the observed *versus* the expected frequency of CPDs, as calculated from the DNA sequence and employing the algorithm described in [45]. There is a very good correlation between the observed and expected frequencies. We also examined whether or not this approach could be employed to detect changes in CPD frequencies due to DNA repair. Figure 17 shows data indicating that it can. Here the change in frequency seen during a 2 h repair period in NER competent cells is compared to that seen with *rad4* NER defective cells. Variable changes are seen when repair operates but no change in frequency is seen in the repair defective mutant.

With respect to the precise kinetics of NER at specific sites within the genome, sufficient experiments are needed to provide statistically meaningful results and internal standards with known levels of DNA damage on the microarrays are required to enable comparisons between arrays. Such refinements of the approach will enable quantitative ChIP on Chip analyses of DNA damage throughout entire genomes alongside concomitant epigenetic changes.

This combination of immunoprecipitation and microarray technology for examining DNA damage will enable researchers to analyse repair events in an entire genome. Analyses of genome-wide DNA repair can be undertaken, along with the examination of the DNA damage-induced changes in chromatin that facilitate repair and how these changes are reversed to return the chromatin to its pre-damaged status. For example, histone acetylations, other covalent histone modifications, the recruitment of specific enzymes such as histone acetyltranferases and histone deacetylases, the changes in nucleosome positions and the chromatin remodelling factors responsible for this. These events can be examined alongside the sequential recruitment of DNA repair enzymes. These sorts of experiments are crucial to identify where in the genome the requirements differ for the chromatin modification enabling efficient NER. The approach can examine other DNA damages provided either the antibodies or the tagged DNA damage recognition enzymes are available to immunoprecipitate those specific damages.

The method is applicable to studies with human cells via the available human arrays. DNA samples prepared for this microarray-based approach can be readily processed for high throughput sequencing analysis if this is preferred, or if microarrays are unavailable for the organism of interest. With respect to human studies, the approach will help investigate whether or not there are polymorphisms for chromatin modifying enzymes that might impinge on NER capacity for only selected genomic regions, and whether these polymorphisms play any roles in events such as cancer risk.

## 3. Experimental Section

### 3.1. Yeast Strains

The haploid yeast strains employed are described in detail in the various publications cited in each of the results sections [7–20,33]. All strains used were stored and grown in Yeast Extract Peptone Dextrose (YPD) media. When long-term storage of the strains was required, cells were grown to exponential phase in YPD with 30% glycerol and after kept at −80 °C. If strains were frequently required they were streaked on YPD with agar solid medium. Pre-cultures were formed by inoculating strains into 10 mL of YPD and incubating at 30 °C with shaking until the cell density reached stationary phase and subsequently stored at 4 °C. Large amounts of cell culture needed for repair experiments (~200 mL/repair time) were obtained by inoculating a pre-calculated volume of pre-culture into YPD. The volume of pre-culture to add depended on the particular strains growth rate. The strains in YPD were generally grown over-night at 30 °C with shaking so as to reach a cell density of 2 × 10^7^ cells/mL.

### 3.2. UV Irradiation of Yeast Cells

Yeast cells were irradiated with UV light in order to induce DNA damage and/or various cellular responses to this agent prior to the extraction of DNA or chromatin. Exponential phase yeast cells were re-suspended in pre-chilled phosphate buffered saline and adjusted with PBS to a density of 2 × 10^7^ cells/mL. Cells were irradiated in 50 mL aliquots with 254 nm of UV light. Prior to UV irradiation an untreated sample was always taken that was not exposed to any UV. The irradiated cells were re-suspended in YPD and allowed to repair for various times at 30 °C in the dark. All samples were kept in the dark to prevent repair by photoreactivation. At the various repair times, the samples were collected by centrifugation at 4 °C.

### 3.3. UV Survival Assays

Yeast cells were grown to late log phase in YPD, counted and then diluted to 2 × 10^7^ cells/mL. The cells were subject to serial dilutions until at a final concentration of 2000 cells/mL. From this 100 μL (200 cells) were pipetted onto 2% agar YPD plates. Plates were then irradiated with a range of UV doses, always including an untreated control. All UV doses were conducted in triplicate. Following UV irradiation, plates were kept in the dark and incubated at 30 °C for 48 h to allow colony formation. To determine UV survival, colonies were counted and the percent of surviving cells was calculated using the untreated average as 100%.

### 3.4. Preparation of Yeast DNA

For yeast DNA extraction, the cells were firstly treated with zymolyase to create spheroplasts, which could then by lysed with a lysis buffer containing sodium dodecyl sulphate (SDS). Following RNase A and Pronase treatment, to digest the RNA and protein respectively, the DNA was extracted using phenol/chloroform and precipitated using ethanol. The maximum number of cells used for genomic DNA isolation was 1 × 10^10^ cells/sample and this yields on average 300–500 μg of genomic DNA from haploid yeast strains. The DNA could be stored at 4 °C short-term or at −20 °C for longer term storage.

### 3.5. Preparation of Yeast Chromatin

Proteins and DNA were cross-linked to each other using 37.5% Formaldehyde, prior to cell lysis using glass beads and shaking. DNA was fragmented using sonication with a Bioruptor to a size of approximately 500 bp. The maximum number of cells used for genomic chromatin isolation was 6 × 10^7^ cells/sample. The full procedure is as follows:

1. UV irradiation of yeast cells took place as described above. However after re-suspension of the cells into YPD 3 mL of 37.5% Formaldehyde was added per 100 mL of culture to form protein-DNA and protein-protein cross-links. The culture underwent incubation on a shaking platform at room temperature for between 10 min and 40 min dependent on the protein of interest. Following this incubation 5.5 mL of 2.5 M glycine was added and incubated at room temperature on a shaking platform for 5 min. This step stopped the crosslinking. All samples were collected by centrifugation and re-suspended in approximately 40 mL PBS, where-after the cells were kept on ice and in the dark.
2. Cells in 40 mL PBS were pelleted, before washing and transferring to a 2 mL microcentrifuge tube using 1 mL of FA/SDS. The cells were pelleted at 4 °C, the supernatant was removed and the pellet was re-suspended in 500 μL FA/SDS( + PMSF).
3. Mechanical lysing of the cell wall occurred by adding 0.5 mL of glass beads to each sample, before vortexing for 10 min at 4 °C. The chromatin was purified from the beads by puncturing a hole in the 2 mL microcentrifuge tube with a hot needle prior to the placement of the microcentrifuge tube in a 15 mL Falcon tube.
4. The lysate was collected in the 15 mL Falcon tube by centrifugation, before the glass beads were washed with 500 μL of FA/SAS buffer (+PMSF) and subjected to a further centrifugation.
5. The cell lysate should have exited the microcentrifuge tube through the hole generated and have collected in the 15 mL Falcon tube. In order to remove any soluble proteins not crosslinked to the DNA, the cell lysate was transferred to a 2 mL microcentrifuge tube and centrifuged at 12,000 rpm for 20 min at 4 °C in a microfuge (Beckman Coulter, 22R centrifuge). The supernatant was removed by aspiration and the remaining pellet re-suspended and transferred to a 15 mL Corning tube with 900 μL FA/SDS (+PMSF).
6. Sonication was carried out using a Bioruptor, set to High and at 4 °C, for 20 s ON followed by 40 s OFF. Metal probes were cleaned with 100% ethanol and then with H_2_O before being screwed into the 15 mL Corning tubes and placed in the Bioruptor water bath. The settings on the Bioruptor were checked, and the programme started for 6 cycles.
7. Following sonication, the 15 mL Corning tubes were centrifuged at 4000 rpm for 10 min. The supernatant was transferred to a 1.5 mL microcentrifuge tube and centrifuged at 12,000 rpm in a microfuge for 20 min.
8. Finally the supernatant was transferred to a fresh 1.5 mL microcentrifuge tube. The 1 mL of chromatin was snap frozen with liquid nitrogen and stored at −80 °C DNA gel electrophoresis.

DNA electrophoresis in agarose gel was routinely used in this study for monitoring DNA quality and relative quantity, for establishing DNA manipulations had taken place, for the analysis of CPDs at the level of the gene by Southern blotting, and for the analysis and quantification of CPDs at nucleotide resolution. The details can be obtained from our various publications which we cite [7–20].

### 3.6. Chromatin Immunoprecipitation (ChIP)

Chromatin prepared as described earlier was subjected to immunoprecipitation using specific antibodies that were linked to magnetic Dynabeads and which had been raised against the protein of interest or against CPDs. The antibody and Dynabeads serve to pull down or immunoprecipitate the DNA fragments where the protein of interest has bound (an immunoprecipitated or IP sample). In addition a control sample is obtained which is subjected to no immunoprecipitation (input sample). Following from this for samples where we examined histones in chromatin both IP and input samples undergo cross-link reversal, pronase and RNase digestion before DNA purification. The procedure is as follows:

1. For each sample to be IP’d, 50 μL of Dynabeads (anti-Rabbit Ig G, Invitrogen) was taken and washed 3 times with 500 μL PBS-BSA (0.1%). After the washes, the Dynabeads were re-suspended in 100 μL PBS-BSA (0.1%) per sample before the addition of a specific antibody. An antibody titration experiment was performed beforehand to determine the amount of antibody to add per sample for optimal immunoprecipitation. For this protocol, usually 2 μg of antibody per sample was required.
2. The mixture of Dynabeads and antibody was incubated at 30 °C, for 30 min at 1300 rpm in an Eppendorf thermomixer. At this stage the antibody should attach to the Dynabeads.
3. The Dynabeads were collected using a magnet held against the tube, and washed 3 times with 500 μL PBS-BSA (0.1%). Finally the Dynabeads were re-suspended in 50 μL of PBS-BSA (0.1%) per sample. From this master mix, 50 μL of beads was added to 100 μL of sonicated chromatin. In addition 30 μL 10× PBS-BSA (10 mg/mL) was added and the final volume adjusted to 300 μL in total with PBS. This was incubated at 21 °C at 1300 rpm for 3 h in an Eppendorf Thermomixer.
4. After the incubation, the Dynabeads (plus antibody plus DNA) were collected using a magnet and the supernatant removed. The beads were washed with 500 μL FA/SDS buffer. This was followed by a series of washes: 3 washes with 1 mL FA/SDS + NaCl buffer; 1 wash with 500 μL LiCl buffer and finally with 500 μL cold TE.
5. After the final wash, the DNA was eluted off the Dynabeads with 125 μL of Pronase buffer at 65 °C, at 900 rpm for 20 min in an Eppendorf Thermomixer. Following this the supernatant was transferred to a fresh 1.5 mL microcentrifuge tube and 6.25 μL of Pronase was added before incubation overnight at 65 °C in a water bath.
6. For the input samples, 50 μL of sonicated chromatin was adjusted to 100 μL with TE buffer, before the addition of 25 μL 5× Pronase Buffer. Like the IP samples, the input samples all had 6.25 μL of Pronase added and were incubated overnight at 65 °C in a water bath.
7. The following day, the IP and input samples were all treated with 2 μL of RNase and incubated for 1 h at 37 °C. After the incubation, the DNA was purified using Qiagen PCR purification kit (followed as per manufacturer’s instructions) into 50 μL elution buffer. From this stage the IP and input DNA could either be used in a qRT-PCR or used as the starting point for genome-wide experiments.

For the analysis of UV induced CPDs throughout the yeast genome the procedures described in [45] were employed.

### 3.7. Quantitative Real-Time PCR (qRT-PCR)

qRT-PCR was used to determine the relative amounts of DNA immunoprecipitated between different repair times. The precipitated DNA and input DNA was amplified by PCR using the iQTM SYBRgreen supermix (BioRad) and analysed using the iCycler iQ real-time PCR detection system (BioRad). SYBRgreen dye can bind to dsDNA and strongly fluoresces when bound, the fluorescence is proportional to the amount of DNA. Thus when the amount of DNA amplifies during the PCR, so does the corresponding fluorescence value. The fluorescence is measured during each PCR cycle, during the exponential phase of PCR amplification. All data was analysed using the iCycler software (BioRad). During each experiment a set of standards is included; from the fluorescence values of the standards a relative value for the dsDNA amount in each of the samples being assayed can be determined. Data analysis was carried out using iCycler software (BioRad). Ct is inversely proportional to the copy number of the target template. Thus a low Ct value represents a high starting concentration and *vice versa*. The amount of DNA is determined using the results of the standard curve produced from either a serial dilution of DNA with a known concentration or serial dilution of DNA given an arbitrary concentration.

### 3.8. Preparation of Yeast Whole Cell Extract (WCE)

Yeast cells were grown overnight in YPD at 30 °C in a shaking incubator until they reached a density of 2 × 10^7^ cells/mL. Cells could be UV irradiated at this stage as described earlier. Cells were pelleted and re-suspended back into 50 mL YPD per repair time and into a fresh 50 mL Falcon tube. The 50 mL sample was centrifuged to pellet the cells and the supernatant was removed. The pellet was re-suspended and transferred to a 2 mL microcentrifuge tube using 1 mL of chilled PBS. When all samples where in 1 mL PBS, the 2 mL microcentrifuge tubes were centrifuged at 4 °C at 13,000 rpm for 8 min. The supernatant was aspirated and the pellet re-suspended in 500 μL Yeast Dialysis Buffer (20 mM Hepes-KOH, 0.01 M EDTA, 0.01 M MgSO_4_, 10% Glycerol, 5 mM DTT and 1× protein inhibitors).

Mechanical breakage of cell wall was undertaken by addition of 0.5 mL glass beads and vortexing tubes at 4 °C for 2 min, 1 min rest on ice, repeated 4 times. Following this the tubes were centrifuged at 13,000 rpm at 4 °C for 15 min. The supernatant was collected into fresh 1.5 mL microcentrifuge tubes. The protein content could be quantified using the Bradford assay (BioRad). WCEs were frozen with liquid nitrogen and stored at −80 °C.

## 4. Conclusions

In conclusion, we have summarised our current knowledge of the relationships between histone acetylation and NER in the chromatin of *Saccahromyces cerevisiae*. We have also outlined how recent technical developments now enable entire genomes to be examined. These developments mean that we will soon have a comprehensive view as to how chromatin is modified for efficient NER throughout the yeast genome and how it is restored to its pre-damaged status to maintain epigenetic codes. The research with yeast is rapidly progressing and because these technologies are equally applicable to studies with the more complex human genome, we will no doubt be unravelling relationships between these events and human genome stability in the near future.

## References

[1] Ramanathan B., Smerdon M.J. (1986). Changes in nuclear protein acetylation in UV-damaged human cells. Carcinogenesis.

[2] Ramanathan B., Smerdon M.J. (1989). Enhanced DNA repair synthesis in hyperacetylated nucleosomes. J. Biol. Chem.

[3] Smerdon M.J., Thoma F. (1990). Site-specific DNA repair at the nucleosome level in a yeast minichromosome. Cell.

[4] Wellinger R.E., Thoma F. (1997). Nucleosome structure and positioning modulate nucleotide excision repair in the non-transcribed strand of an active gene. EMBO J.

[5] Friedberg E.C., Walker G.C., Siede W., Wood R.D., Schultz R.A., Ellenberger T (2005). DNA Repair and Mutagenesis.

[6] Li S., Waters R. (1996). Nucleotide level detection of cyclobutane pyrimidine dimers using oligonucleotides and magnetic beads to facilitate labeling of DNA fragments incised at dimers and chemical sequencing reference ladders. Carcinogenesis.

[7] Teng Y., Li S., Waters R., Reed S.H. (1997). Excision repair at the level of the nucleotide in the *Saccharomyces cerevisiae* MFA2 gene: The mapping of where enhanced repair in the transcribed strand begins or ends and the identification of only a partial Rad16 requisite for repairing upstream control sequences. J. Mol. Biol.

[8] Teng Y., Longhese M.P., McDonough G., Waters R. (1998). Mutants with changes in different domains of yeast replication protein A exhibit differences in repairing the control region, the transcribed strand and the non-transcribed strand of the *Saccharomyces cerevisiae MFA2* gene. J. Mol. Biol.

[9] Teng Y., Waters R. (2000). Excision repair at the level of the nucleotide in the upstream control region, the coding sequence and the region where transcription terminates of the *S. cerevisiae MFA2* gene and the role of the *RAD26*. Nucleic Acids Res.

[10] Yu S., Teng Y., Lowndes N.F., Waters R. (2001). RAD9, RAD24, RAD16 and RAD26 are required for the inducible nucleotide excision repair of UV-induced cyclobutane pyrimidine dimers from the transcribed and non transcribed regions of the *Saccharomyces cerevisiae MFA2* gene. Mutat. Res.

[11] Teng Y., Yu Y., Waters R. (2002). The *Saccharomyces cerevisiae* histone acetyltransferase Gcn5 has a role in the photoreactivation and nucleotide excision repair of UV induced cyclobutane pyrimidine dimers in the *MFA2* gene. J. Mol. Biol.

[12] Ferreiro J.A., Powell N.G., Karabetsou N., Kent N.A., Mellor J., Waters R. (2004). Cbf1p modulates chromatin structure, transcription and repair at the *S. cerevisiae MET16* locus. Nucleic Acids Res.

[13] Tremblay M., Teng Y., Paquette M., Waters R., Conconi A. (2008). Complementary roles of yeast Rad4p and Rad34p in nucleotide excision repair of active and inactive rDNA chromatin. Mol. Cell Biol.

[14] Taschner M., Harreman M., Teng Y., Gill H., Anindya R., Maslen S.L., Skehel J.M., Waters R., Svejstrup J.Q. (2010). A role for checkpoint kinase-dependent Rad26 phosphorylation in transcription-coupled DNA repair in Saccharomyces cerevisiae. Mol. Cell Biol.

[15] Teng Y., Yu S., Waters R. (2001). The mapping of nucleosomes and regulatory protein binding sites on the *Saccharomyces cerevisiae* MFA2 gene: A high resolution approach. Nucleic Acids Res.

[16] Yu Y., Teng Y., Reed S.H., Waters R. (2005). UV irradiation stimulates histone acetylation and chromatin remodeling at a repressed yeast locus. Proc. Natl. Acad. Sci. USA.

[17] Teng Y., Yu Y., Ferreiro J.A., Waters R. (2005). Histone acetylation, chromatin remodeling, transcription and nucleotide excision repair in *S. cerevisiae*: studies with two model genes. DNA Repair.

[18] Ferreiro J.A., Powell N.G., Karabetsou N., Mellor J., Waters R. (2006). Roles for Gcn5p and Ada2p in transcription and nucleotide excision repair at the *Saccharomyces cerevisiae MET16* gene. Nucleic Acids Res.

[19] Teng Y., Liu H., Gill H.W., Yu Y., Waters R., Reed S.H. (2008). *Saccharomyces cerevisiae* Rad16 mediates ultraviolet-dependent histone H3 acetylation required for efficient global genome nucleotide-excision repair. EMBO Rep.

[20] Yu S., Teng Y., Waters R., Reed S.H. (2011). How chromatin is remodeled during DNA repair of UV-induced DNA damage in *Saccharomyces cerevisiae*. PLoS Genet.

[21] Flaus A., Owen-Hughes T. (2011). Mechanisms for ATP-dependent chromatin remodeling: The means to the end. FEBS J.

[22] Ferreira H., Flaus A., Owen-Hughes T. (2007). Histone modifications influence the action of Snf2 family remodeling enzymes by different mechanisms. J. Mol. Biol.

[23] Svejstrup J.Q. (2002). Mechanisms of transcription-coupled DNA repair. Nat. Rev. Mol. Cell. Biol.

[24] Li S., Smerdon M.J. (2002). Rpb4 and Rpb9 mediate subpathways of transcription-coupled DNA repair in *Saccharomyces cerevisiae*. EMBO J.

[25] Verhage R., Zeeman A.M., de Groot N., Gleig F., Bang D.D., van de Putte P., Brouwer J. (1994). The *RAD7* and *RAD16* genes, which are essential for pyrimidine dimmer removal from the silent mating type loci, are also required for repair of the nontranscribed strand of an active gene in *Saccharomyces cerevisiae*. Mol. Cell. Biol.

[26] Reed S.H., Akiyama M., Stillman B., Friedberg E.C. (1999). Yeast autonomously replicating sequence binding factor is involved in nucleotide excision repair. Genes Dev.

[27] Yu S., Smirnova J.B., Friedberg E.C., Stillman B., Akiyama M., Owen-Hughes T., Waters R., Reed S.H. (2009). ABF1 binding sites promote efficient global genome nucleotide excision repair. J. Biol.Chem.

[28] Bang D.D., Verhage R., Goosen N., Brouwer J., van de Putte P. (1992). Molecular cloning of *RAD16*, a gene involved in differential repair in Saccharomyces cerevisiae. Nucleic Acids Res.

[29] Eisen J.A., Sweder K.S., Hanawalt P.C. (1995). Evolution of the SNF2 family of proteins: Subfamilies with distinct sequences and functions. Nucleic Acids Res.

[30] Whitehouse I., Flaus A., Cairns B.R., White M.F., Workman J.L., Owen-Hughes T. (1999). Nucleosome mobilization catalysed by the yeast SWI/SNF complex. Nature.

[31] Havas K., Flaus A., Phelan M., Kingston R., Wade P.A., Lilley D.M., Owen-Hughes T. (2000). Generation of superhelical torsion by ATP-dependent chromatin remodeling activities. Cell.

[32] Van Komen S., Petukhova G., Sigurdson S., Stratton S., Sung P. (2000). Superhelicity-driven homologous DNA pairing by yeast recombination factors Rad51 and Rad54. Mol. Cell.

[33] Yu S., Owen-Hughes T., Friedberg E.C., Waters R., Reed S.H. (2004). The yeast Rad7/Rad16/Abf1 complex generates superhelical torsion in DNA that is required for nucleotide excision repair. DNA Repair.

[34] Wang Z., Wei S., Reed S.H., Wu X., Svejstrup J.Q., Feaver W.J., Kornberg R.D., Friedberg E.C. (1997). The *RAD7*, *RAD16* and *RAD23* genes of *Saccharomyces cerevisiae*: Requirement for transcription-independent nucleotide excision repair *in vitro* and interactions between the gene products. Mol. Cell Biol.

[35] Guzder S.N., Sung P., Prakash L., Prakash S. (1997). Yeast Rad7-Rad16 complex, specific for the nucleotide excision repair of the nontranscribed DNA strand, is an ATP-dependent DNA damage sensor. J. Biol. Chem.

[36] Gillette T.G., Yu S., Zhou Z., Waters R., Johnston S.A., Reed S.H. (2006). Distinct functions of the ubiquitin-proteasome pathway influence nucleotide excision repair. EMBO J.

[37] Diffley J. (1992). Early events in eukaryotic DNA replication. Trends Cell Biol.

[38] Yarragudi A., Parfrey L.W., Morse R.H. (2007). Genome-wide analysis of transcriptional dependence and probable target sites for *Abf1* and *Rap1* in *Saccharomyces cerevisiae*. Nucleic Acids Res.

[39] Bone J.R., Roth S.Y. (2001). Recruitment of the yeast Tup1p-Ssn6p repressor is associated with localized decreases in histone acetylation. J. Biol. Chem.

[40] Malavé T.M., Dent S.Y. (2006). Transcriptional repression by Tup1-Ssn6. Biochem. Cell Biol.

[41] Irizar A., Yu Y., Reed S.H., Louis E.J., Waters R. (2010). Silenced yeast chromatin is maintained by Sir2 in preference to permitting histone acetylations for efficient NER. Nucleic Acids Res.

[42] Loney E.R., Inglis P.W., Sharp S., Pryde F.E., Kent N.A., Mellor J., Louis E.J. (2009). Repressive and non-repressive chromatin at native telomeres in *Saccharomyces cerevisiae*. Epigenet. Chromatin.

[43] Mockler T.C., Chan S., Sundaresan A., Chen H., Jacobsen S.E., Ecker J.R. (2005). Applications of DNA tiling arrays for whole-genome analysis. Genomics.

[44] Pokholok D.K., Harbison C.T., Levine S., Cole M., Hannett N.M., Lee T.I., Bell G.W., Walker K., Rolfe P.A., Herbolsheimer E. (2005). Genome-wide map of nucleosome acetylation and methylation in yeast. Cell.

[45] Teng Y., Bennett M., Evans K.E., Zhuang-Jackson H., Higgs A., Reed S.H., Waters R. (2011). A novel method for the genome-wide high resolution analysis of DNA damage. Nucleic Acids Res.

